# Working Memory in the Prefrontal Cortex

**DOI:** 10.3390/brainsci7050049

**Published:** 2017-04-27

**Authors:** Shintaro Funahashi

**Affiliations:** Kokoro Research Center, Kyoto University, Yoshida, Sakyo-ku, Kyoto 606-8501, Japan; funahashi.shintaro.35e@st.kyoto-u.ac.jp; Tel./Fax: +81-77-544-5106

**Keywords:** Prefrontal cortex, working memory, reference memory, monkey, central executive, delay-period activity, dual task, metamemory

## Abstract

The prefrontal cortex participates in a variety of higher cognitive functions. The concept of working memory is now widely used to understand prefrontal functions. Neurophysiological studies have revealed that stimulus-selective delay-period activity is a neural correlate of the mechanism for temporarily maintaining information in working memory processes. The central executive, which is the master component of Baddeley’s working memory model and is thought to be a function of the prefrontal cortex, controls the performance of other components by allocating a limited capacity of memory resource to each component based on its demand. Recent neurophysiological studies have attempted to reveal how prefrontal neurons achieve the functions of the central executive. For example, the neural mechanisms of memory control have been examined using the interference effect in a dual-task paradigm. It has been shown that this interference effect is caused by the competitive and overloaded recruitment of overlapping neural populations in the prefrontal cortex by two concurrent tasks and that the information-processing capacity of a single neuron is limited to a fixed level, can be flexibly allocated or reallocated between two concurrent tasks based on their needs, and enhances behavioral performance when its allocation to one task is increased. Further, a metamemory task requiring spatial information has been used to understand the neural mechanism for monitoring its own operations, and it has been shown that monitoring the quality of spatial information represented by prefrontal activity is an important factor in the subject's choice and that the strength of spatially selective delay-period activity reflects confidence in decision-making. Although further studies are needed to elucidate how the prefrontal cortex controls memory resource and supervises other systems, some important mechanisms related to the central executive have been identified.

## 1. Introduction

The prefrontal cortex participates in a variety of higher cognitive functions, such as thinking, reasoning, planning, and decision-making [1,2,3,4,5]. Therefore, the prefrontal cortex is thought to be an essential brain area for examining the origin of human intelligence and creativity. This idea has been supported by several lines of evidences. For example, in humans, the prefrontal cortex occupies 29% of the cerebral cortex [4], which is the largest percentage among primate species and indeed mammalian species. The prefrontal cortex takes a longer time to mature than other association cortices [4], suggesting that the prefrontal cortex does not participate in basic sensory or motor information processing, but rather participates in more complex and highly integrated functions. This notion is supported by the fact that the prefrontal cortex receives all kinds of sensory information from sensory association cortices, but not from the primary sensory cortices [6,7,8,9,10], and the prefrontal cortex sends motor information to motor association cortices, such as the supplementary motor area and the premotor cortex [11,12]. The importance of the prefrontal cortex in controlling cognitive functions is also supported by the finding that damage to the prefrontal cortex produces poor judgment, planning, and decision-making in humans [13].

Although the prefrontal cortex is thought to participate in important cognitive functions in humans, little is known about the mechanism by which the prefrontal cortex produces these functions. However, studies using nonhuman primates have contributed significantly to our understanding of prefrontal functions and their neural mechanisms. Since Jacobsen (1936) [14] first reported that rhesus monkeys with bilateral prefrontal lesions exhibited severe and long-lasting impairment of delayed-response performance, the delayed-response task has become an essential behavioral task for examining prefrontal functions experimentally using animals. Many important observations have been made using this task [4,15,16]. Jacobsen (1936) [14] initially suggested that delayed-response deficits were caused by an impairment of the mechanism for the short-term storage of spatial information. However, the true nature of the delayed-response deficit was not clear and there was some disagreement regarding the cause of prefrontal deficits in animal lesion studies and human clinical studies. Goldman-Rakic (1986) [1] proposed that the prefrontal deficits observed in both monkey lesion studies and human clinical studies can be explained by using a common concept of working memory. Although she originally used the term “representational memory”, not working memory, she later used working memory to describe the ability to hold information transiently in mind in the service of comprehension, thinking, and planning [17,18]. After this proposal, the idea that working memory is a key concept for understanding prefrontal functions has been supported by numerous human neuroimaging studies and animal studies.

The concept of working memory was first proposed by Baddeley and Hitch (1974) [19] and has been widely used in human psychology. Working memory has also been used in animal studies. In animal studies, working memory was originally used to explain hippocampal functions [20,21,22,23,24,25,26]. However, working memory has now become the most important concept for interpreting and understanding prefrontal cortical functions in both humans and animals. Although Baddeley’s model of working memory has been widely used to explain the results in human psychological and neuropsychological studies, it is difficult to adapt this model for animal studies. In this review, I will first describe how working memory has been defined in human studies and animal studies. Since a common concept is that working memory is a unique short-term active storage mechanism, I will then explain how information is actively maintained in the neural system based on prefrontal studies using primates. Among the four components of the working memory model that Baddeley proposed, the central executive plays the most important role and the prefrontal cortex has been considered to play this function. Therefore, it is important to consider how the prefrontal cortex can achieve this function. Recently, neural mechanisms for the flexible allocation of memory and neural mechanisms that support metamnemonic functions have been examined in the prefrontal cortex. These two functions are closely related to the functions of the central executive. I will discuss the importance of these studies to understand the neural mechanisms of working memory and the central executive.

## 2. Working Memory

### 2.1. Working Memory in Human Studies

The term “working memory” describes a unique short-term active storage mechanism that is used to achieve a variety of cognitive activities including thinking, reasoning, judging, decision-making, and language comprehension. Working memory is sometimes differentiated from short-term memory, such that short-term memory refers to the simple temporary storage of information, whereas working memory refers to both the storage and manipulation of information. Atkinson and Shiffrin (1968) [27] first introduced a model of short-term memory and explained that this short-term memory acted as working memory when the subject performed a variety of cognitive activities. Subsequently, Baddeley and Hitch (1974) [19] first proposed the concept of working memory. They defined working memory as a brain system that provides for the temporary storage and manipulation of information that is necessary for complex cognitive tasks such as language comprehension, learning and reasoning. Baddeley and Logie (1999) [28] further described working memory as a mechanism that allowed humans to comprehend and mentally represent their immediate environment, retain information about their immediate past experience, support the acquisition of new knowledge, solve problems, and formulate, relate, and act on current goals. Similarly, Kieras et al. (1999) [29] described working memory as a mechanism for encompassing the entire ensemble of temporary stored codes, knowledge representations, and procedures whereby information is maintained, updated, and applied for performing perceptual-motor and cognitive tasks. Further, Miyake and Shah (1999) [30] described working memory as the mechanisms or processes that are involved in the control, regulation, and active maintenance of task-relevant information in the service of complex cognition, including both novel and familiar skilled tasks. Thus, although working memory is classified as a kind of short-term memory, it consists of neural processes for the short-term active maintenance and manipulation of information. Working memory is thought to be an important concept for understanding neural mechanisms for a variety of higher cognitive functions.

Several models of working memory have been proposed [30]. The most influential model of working memory was the one proposed by Baddeley and Hitch (1974) [19] and Baddeley (1986) [31]. Baddeley’s original model of working memory included one master component (the central executive) and two slave components (the phonological loop and the visuo-spatial sketchpad). The phonological loop is a system for speech perception and language comprehension. This component includes mechanisms for the temporary storage of speech-based information. Although subvocal rehearsal was assumed to be necessary for the maintenance of information, storage—rather than the rehearsal of information—was emphasized. On the other hand, the visuo-spatial sketchpad is a system for processing visuo-spatial information and information that cannot be processed by language. The visuo-spatial sketchpad includes mechanisms for the temporary storage of visual images and spatial information. Although the original model of working memory had two slave components, Baddeley (2000) [32] proposed a revised model of working memory that included an additional slave component (the episodic buffer). The episodic buffer is a temporary storage buffer with a limited capacity and has functions to integrate information that arise from a variety of sources including long-term memory. The episodic buffer is assumed to hold integrated episodes or chunks and not only acts as a buffer between the phonological loop and the visuo-spatial sketchpad, but also links working memory with perception and long-term memory. Thus, the three slave components include mechanisms for the temporary storage of information: the phonological loop temporarily stores speech-based information, the visuo-spatial sketchpad temporarily stores visual images and spatial information, and the episodic buffer temporarily stores integrated episodes and chunks from perception and long-term memory. On the other hand, Baddeley (1986, 2000) [31,32] proposed the central executive as the master component for coordinating and integrating the operations of these three slave components to achieve a current goal. The central executive is thought to be a system for allocating a limited capacity of memory resource to each of the slave components based on their demands. If visuo-spatial processing becomes more demanding than language processing, more memory will be allocated to visuo-spatial processing. Thus, an appropriate control process or strategy would eventually be selected to accomplish a current goal. Recently, Baddeley (2012) [33] proposed a revised model of working memory, but did not change its basic structure.

### 2.2. Working Memory in Animal Studies

The term “working memory” was originally introduced in human memory studies [31]. However, this term had also been used in animal studies. In animal studies, “working memory” was usually distinguished from “reference memory” [34,35,36,37,38,39,40]. In animal studies, “working memory” was defined as a memory system that retains information that is necessary for only one trial, not for subsequent trials, while “reference memory” was considered to be a memory that was useful over many trials and even for an entire experiment that lasted for several weeks or months [22,41]. The visual discrimination task is an example of a task that is considered to require reference memory. In this task, the subject’s behavioral response to a particular visual stimulus is always rewarded throughout the experiment. Therefore, the formation of an association between a particular visual stimulus and a particular response is required for correct performance, and long-term maintenance of this association is necessary. The essential factors in the reference memory task are the formation of a particular association between a stimulus and a response by learning and long-term maintenance of this association, which are similar to those that are essential for establishing long-term memory in humans. Therefore, behavioral tasks that require reference memory have often been used to examine the neural mechanisms of long-term memory in animal studies [22].

On the other hand, “working memory” was defined as memory that retains information that is necessary only for the current trial, not for subsequent trials [22,41]. The critical difference between a working memory task and a reference memory task is that, in the former, different stimuli guide different responses in different trials. In working memory tasks, the cue information that the subject needs to remember varies from trial to trial. Therefore, the formation of an association between a particular stimulus and a particular response or learning of a stimulus-response association, which are essential for reference memory tasks, is not required. The delayed-response task and the delayed-alternation task are examples of working memory tasks, in which the subject is required to maintain spatial information, such as the location of the bait, during the delay period. The delayed matching-to-sample task and the delayed non-matching-to-sample task are other examples of working memory tasks in which the subject is required to maintain non-spatial information, such as an object itself, its shape, or its color, during a delay period.

In the delayed-response task using monkeys, first, the reward is placed in one of two food wells. Next, the food wells are covered with identical opaque plates so that the subject cannot see where the reward is hidden. After a delay period of several seconds, the subject is required to select the correct food well. Since the location of the bait varies randomly from trial to trial, the subject needs to remember the spatial information specific for the bait location and use this information to make a correct choice. Similarly, in the delayed-alternation task, the subject needs to select the food well alternately. Therefore, the subject is required to maintain information regarding which food well it selected in the preceding trial. Since the information maintained during the delay period is useful only for the current trial and since both tasks require the short-term storage of the information regarding the baited position, these tasks are called “spatial” working memory tasks [1,25,42]. Both the delayed-response task and delayed-alternation task are used not only in primate studies, but also in rat and mouse studies.

On the other hand, the delayed matching-to-sample task and the delayed non-matching-to-sample task are examples of non-spatial working memory tasks [25,36,40,43]. In these tasks, a sample stimulus is first presented briefly, and then a delay period is introduced for several seconds to minutes. After the delay period, the sample stimulus and another stimulus are presented simultaneously and the subject needs to select the sample stimulus (delayed matching-to-sample task) or the other stimulus (delayed non-matching-to-sample task) to get a reward. The information that is temporarily maintained during the delay period is a non-spatial visual feature such as the object itself, its shape, its pattern, or its color. This information is useful only for the current trial, and a different stimulus is used as the sample stimulus in the next trial. Therefore, these tasks are also classified as working memory tasks.

However, these tasks are also called “recognition memory” tasks in monkey studies [44,45,46,47,48,49,50]. Since in these tasks, one item is presented as a sample stimulus and, after a delay period, the same item is presented again together with other items, the subject can choose a correct item not only based on their memory of the sample item itself, but also based on the familiarity or the sameness of the item. Therefore, classification based on the fact that the item is “familiar or unfamiliar” or “same or different” can lead to the correct response in these tasks.

Although both delayed-response tasks and recognition memory tasks are considered to be types of working memory tasks, an important difference between these two tasks is that, in the former, information that might suggest the correct location of the bait is not available to the subject during the response period, while in the latter, the same stimulus that was presented during the sample period is presented again together with other stimuli during the choice period. The only essential factor that is required to perform the delayed-response task correctly is memory of the location of the bait, not the familiarity or sameness of the stimulus or the formation of a stimulus-response association. On the other hand, in recognition memory tasks, the subject can select the correct item not only based on the memory of the sample item itself, but also based on the familiarity or sameness of the item. Therefore, recognition memory tasks may be distinct from delayed-response tasks. Recognition memory tasks have often been used to examine mnemonic functions of the hippocampus and the medial temporal lobe. It has been shown that monkeys with lesions in these brain areas exhibit deficits in recognition memory tasks [49,50]. On the other hand, the delayed-response task has been widely used to examine prefrontal cortical functions [1,4,15].

## 3. Neural Mechanisms of Spatial Working Memory in the Prefrontal Cortex

Working memory is a unique short-term active storage mechanism that is used to achieve a variety of cognitive activities. Working memory is also a memory system that retains specific information that is necessary only in a particular situation. Although human studies and animal studies involve different behavioral tasks and conditions, they use similar definitions of working memory. To understand the neural mechanisms that support working memory, appropriate behavioral paradigms and appropriate animal models are needed for neurophysiological studies. Although both the delayed-response task and recognition memory tasks are considered to be types of working memory tasks, as an appropriate behavioral task, the delayed-response task offers several advantages over recognition memory tasks [15]. First, the only information that needs to be memorized is the spatial position where the reward was placed or where the visual cue was presented. Therefore, the same reward and the same visual cue can be used repeatedly throughout the experiment. Second, the position of the visual cue is easy to control and change randomly, and can be described with great accuracy. Third, the subject can easily report the position of the visual cue by hand-arm movements or eye movements. If eye movement is used as the response behavior, it is easy to describe and analyze the subject’s behavioral response quantitatively. Fourth, familiarity effects can be excluded from the possible factors that could affect the subject’s behavioral performance. Therefore, the delayed-response task would be an appropriate behavioral task for examining the neural mechanisms that underlie working memory functions. As an appropriate animal model, nonhuman primates have several advantages. Monkeys have a large and distinct prefrontal cortex, which includes all of the corresponding cytoarchitectonic areas that have been identified in the human prefrontal cortex. Many anatomical and neuropsychological studies have been performed. Importantly, it has been shown that bilateral lesions of the lateral prefrontal cortex impaired delayed-response performance in monkeys. Thus, neural mechanisms of working memory can be clarified by neurophysiological studies in the prefrontal cortex using monkeys. Using the delayed-response task, neural correlates of the mechanism for the temporary maintenance of information have been examined in the prefrontal cortex.

### 3.1. Search for Neural Correlates of Working Memory in the Prefrontal Cortex: Historical Overview

Since Jacobsen (1936) [14] first showed that rhesus monkeys with bilateral prefrontal lesions exhibited a severe impairment in delayed-response performance, the delayed-response task has become an important behavioral task for examining prefrontal functions in animals [1,4,5]. Many lesion studies in monkeys have repeatedly confirmed that the dorsolateral prefrontal cortex plays significant roles in delayed-response performance. Butters and Pandya (1969) [51] located an area in the prefrontal cortex that was responsible for delayed-response deficits and showed that the middle one-third of the principal sulcal area is a critical cortical area for performing the delayed-response task correctly. Jacobsen (1936) [14] initially suggested that delayed-response deficits were caused by impairment of the mechanism for the short-term storage of spatial information. Although this idea was criticized by others [52,53], it is still a powerful hypothesis that has been supported by an experiment using an oculomotor version of the delayed-response task [54]. If the impairment of short-term memory is a major cause of the delayed-response deficit, the neural mechanism responsible for the short-term storage of spatial information necessary for performing the delayed-response task must be present in the dorsolateral prefrontal cortex. Neurophysiological studies performed in this cortical area using the delayed-response task may be able to identify the neural correlates of mnemonic processes.

Kubota and Niki (1971) [55] used a delayed-alternation task in which monkeys pressed two keys alternately by hand after a delay period and first reported prefrontal activities related to the performance of this task, such as activation in response to visual cue presentation and differential activation during the response period depending on the direction of the response. Fuster and Alexander (1971) [56] used a delayed-response task under a condition similar to that in the Wisconsin General Test Apparatus, in which food wells were located on the left and the right and monkeys were required to select the correct food well by hand. They reported some important characteristics of prefrontal single-neuron activity. For example, they showed that some prefrontal neurons maintained a higher discharge rate throughout the delay period compared with the discharge rate during the inter-trial interval. Subsequently, Fuster (1973) [57] used the same apparatus for the delayed-response task and showed that, although some neurons were transiently active during visual cue presentation or during the manual response, many prefrontal neurons exhibited memory-related activity, which was tonic sustained activation during the delay period. This tonic sustained activation was observed only in correct trials, and was not observed in error trials or trials in which a reward was not given. However, transient activation during the cue and response periods was observed even in trials in which a reward was not given. For monkeys to perform the delayed-response task correctly, they must maintain spatial information regarding the baited position during the delay period. Therefore, Fuster (1973) [57] suggested that tonic sustained excitation during the delay period is attributable to the role of the prefrontal cortex in short-term mnemonic processes, while transient excitation during the cue and response periods is associated with sensory and motor processes, respectively.

In the delayed-response task, the baited position changes randomly between right and left from trial to trial. Therefore, the subject needs to actively maintain the information regarding the baited position during the delay period. If the tonic sustained activity that Fuster (1973) [57] observed is a neural correlate of mnemonic processes that maintain spatial information during the delay period, this activity should exhibit different patterns of activation depending on the baited position. However, Fuster (1973) [57] did not find different patterns of delay-period activity depending on the baited position. Instead, Niki (1974) [58] first found different patterns of delay-period activity. He used a manual delayed-response task, in which monkeys were required to press either a right or left response window that had been illuminated during the cue period, and reported that some prefrontal neurons exhibited delay-period activity of different magnitudes depending on where the visual cue had been presented. Although only a small proportion of prefrontal neurons exhibited this characteristic, Niki (1974) [58] was the first to show a neural correlate of mnemonic processes that maintained spatial information during the delay period in the prefrontal cortex.

Correct performance of the delayed-response task requires that the subject retain not only spatial information regarding where the visual cue was presented, but also information regarding where the response behavior needed to be directed. Therefore, if differential delay-period activity is a neural correlate of mnemonic processes in the prefrontal cortex, this activity must represent either the location of the visual cue or the direction of the behavioral response. However, it was unclear which information was represented by differential delay-period activity. Niki and Watanabe (1976) [59] attempted to examine this issue. They used four response windows and constructed two delayed-response tasks and a conditional position task with a delay. In one delayed-response task, the visual cue was presented at either the right or left response window, while in another delayed-response task, the visual cue was presented at either the upper or lower response window. In the conditional position task, the monkeys were required to press the right (left) response window when the visual cue was presented at the upper (lower) response window. The rationale behind this experiment was that, if delay-period activity represented spatial information regarding the visual cue, significant delay-period activity would be observed in trials when the visual cue was presented at one (e.g., left) response window in these tasks, regardless of the direction of the behavioral response. On the other hand, if delay-period activity represented the direction of the behavioral response, significant activity would be observed in trials when the behavioral response was directed toward one direction (e.g., upper) in these tasks, regardless of where the visual cue was presented. They compared the spatial selectivity of delay-period activity of the same neuron among these three task conditions and found that 70% of differential delay-period activities encoded the position of the visual cue, whereas the remaining 30% encoded the direction of the behavioral response. Thus, prefrontal neurons exhibited differential delay-period activity, most of which represented spatial information of the visual cue (retrospective information), while some represented the direction of the behavioral response (prospective information).

Since then, directionally selective delay-period activity has been reported in several studies while monkeys performed manual delayed-response tasks and manual delayed-alternation tasks [60,61,62,63,64,65,66]. Although these studies used a two-choice (usually left or right choice) or three-choice (left, center, or right choice) paradigm, they all observed directionally selective delay-period activity. Behavioral studies using monkeys indicated that lesions of the dorsolateral prefrontal cortex, especially the cortex within and surrounding the principal sulcus, produced severe and long-lasing deficits in the delayed-response task and the delayed-alternation task [15,16]. The results of neurophysiological studies have shown that the prefrontal cortex is involved in correct performance of the delayed-response task and confirmed that the prefrontal cortex plays an important role in spatial short-term memory function. However, further experiments were needed.

### 3.2. Delay-Period Activity as a Neural Correlate of the Temporary Storage Process in Working Memory

The finding of differential delay-period activity was important for understanding mnemonic functions of the prefrontal cortex. However, to further prove that the prefrontal cortex plays an important role in spatial mnemonic functions, both behavioral and neurophysiological studies are needed in which visual cues are presented in not just a few positions, but rather in multiple positions with different distances and eccentricities. In addition, more strict control of the subject’s behavior may be needed, especially during the delay period. We should be able to exclude the possibility that subjects maintain orientation toward the correct position using head, body, or eye movement during the delay period. Especially, the subject’s eye movements should be carefully controlled during the delay period. It has been shown that many prefrontal neurons exhibit eye movement-related activities [67,68,69,70,71]. Prefrontal neurons have also been shown to exhibit gaze-related activity [72,73,74], such that the magnitude of gaze-related activity changes depending on the direction of the monkey’s gaze [74]. This “angle-of-gaze” effect is known to affect the magnitude of visual, mnemonic, and motor responses in parietal neurons [75,76,77,78]. If the monkey’s eye movements are not controlled during the delay period and if the monkey tends to maintain gazing at either the right or left correct response window during the delay period, differences in the observed delay-period activity might be a result of the angle-of-gaze effect.

To overcome this weakness in preceding studies, an oculomotor version of the delayed-response task (oculomotor delayed-response task, ODR task) has been widely used [79,80,81,82,83,84,85,86,87,88,89,90]. In the ODR task, the subject’s head is immobilized. To control the subject’s eye movements during the delay period, the subject is required to maintain gazing at the central fixation target during this period. This task allows us to easily present visual cues at multiple positions in the visual field. In addition, the locations of visual cues and the trajectories of eye movements can be drawn on the same two-dimensional space.

While monkeys performed the ODR task, delay-period activity was observed in many dorsolateral prefrontal neurons [79]. Delay-period activity showed tonic sustained excitation or suppression during the delay period. Delay-period activity was observed only in correct trials in which the monkey received a reward, and was not observed or was truncated in error trials. In addition, tonic sustained delay-period activity was maintained throughout the delay period. The duration of delay-period activity changed depending on the length of the delay period. These features of delay-period activity support the idea that delay-period activity is a neural correlate of mnemonic processes to temporarily hold information. Notably, a great majority (80%) of delay-period activity exhibited directional selectivity, such that delay-period activity was observed only when visual cues were presented at a particular area in the visual field. The distribution of the best directions of delay-period activity, for which neurons exhibited the maximum discharge, revealed that, although all possible directions were represented, the best directions showed a contralateral bias, such that most delay-period activities had best directions toward the visual field contralateral to the hemisphere where the neurons were located. In addition, significant delay-period activity was observed when the visual cue was presented within a certain area of the visual field. These features of delay-period activity strengthened the idea that delay-period activity is a neural correlate of mnemonic processes to temporarily hold information and indicate that prefrontal neurons with directional delay-period activity have mnemonic receptive fields (memory fields) within the visual field, similar to the finding that neurons with visual responses have visual receptive fields [79,91].

If delay-period activity is a neural correlate of mnemonic processes to temporarily hold information, the next question is what information is maintained during the delay period by delay-period activity. Niki and Watanabe (1976) [59] found that a majority of delay-period activity encoded the position of the visual cue. Similarly, Funahashi et al. (1993b) [92] examined the same issue using two delayed saccade tasks (delayed pro-saccade task and delayed anti-saccade task) and showed that most directional delay-period activity encoded the direction of the visual cue. To further confirm these observations, Takeda and Funahashi (2002) [88] used the original ODR task with 8 cue positions and a rotatory ODR task with 4 cue positions, in which monkeys were required to make a saccade 90° clockwise to the direction where the visual cue had been presented. They compared the best directions of delay-period activity between the two tasks for each neuron. Their results indicated that 86% of directional delay-period activity encoded the position of the visual cue, whereas 13% encoded the direction of the saccade. Thus, most delay-period activity encoded the position of the visual cue, while some encoded the direction of the response behavior. The result that more prefrontal delay-period activities encoded a sensory attribute was also observed in experiments using other tasks. For example, Sawaguchi and Yamane (1999) [93] used a delayed matching-to-space task and found that 90% of prefrontal neurons showed selectivity to the stimulus position, not to the response behavior (either a go or no-go response). Constantinidis et al. (2001a) [85] used an ODR task, in which monkeys were required to make a saccade to the brighter visual cue, and found that a population of prefrontal neurons maintained the sensory attributes of the visual cue throughout the delay period. Thus, these results further support that most prefrontal neurons hold information regarding retrospective sensory attribute during the delay period.

## 4. Importance of Delay-Period Activity in Working Memory

Delay-period activity has been observed in prefrontal neurons while monkeys performed behavioral tasks with a delay period. This activity often exhibits tonic sustained activation during the delay period. However, this activity can also exhibit tonic sustained suppression, or a gradually increasing, or gradually decreasing pattern. Since the definition of delay-period activity is activity observed during the delay period, delay-period activity can be observed in any brain area while the subject performs any behavioral task with delay. In fact, delay-period activity has been observed in the parietal cortex [83,94,95,96,97,98,99,100,101,102,103,104,105], the temporal cortex [106,107,108,109,110,111,112], the somatosensory cortex [113,114], and the premotor cortex [115,116,117,118]. Delay-period activity has been observed in the primary visual cortex [119,120], the superior colliculus [121,122], the basal ganglia [123,124,125,126], the hippocampus [127,128], the thalamus [129,130], and even the spinal cord [131].

Although delay-period activity can be observed in any task with a delay, the information that must be maintained during the delay period can differ from task to task. Therefore, the information that neurons hold as delay-period activity can be different in different tasks. For example, when a monkey performs a delayed-response task, delay-period activity in the prefrontal cortex represents either the location of the visual cue or the direction of the response. In contrast, when a monkey performs a delayed matching-to-sample task, delay-period activity represents non-spatial physical features of the visual stimuli (e.g., color, shape). Prefrontal delay-period activity has been reported to represent a variety of information, such as tactile information [132], auditory information [133], task rules [134,135,136,137], task differences [138], expected reward [139,140,141,142], a numerical quantity [143,144], the relative distance between stimuli [145], timing [146], and the temporal order of stimuli [66], depending on the requirements of the task. This evidence strongly supports the notion that delay-period activity is an important component for understanding the neural mechanisms of working memory.

In addition, different brain areas participate in different functions and different types of information processing. Therefore, the information that neurons hold as delay-period activity is different in different brain areas. For example, in the prefrontal cortex, most delay-period activity encoded the location of the visual cue, while some encoded the direction of the response [59,88,92]. On the other hand, in the parietal cortex, most delay-period activity encoded the direction of the saccade response [77,94,98,100] or the direction of the arm response [95], although delay-period activity encoding sensory stimuli has also been reported [96,102,147] Similarly, in the thalamic mediodorsal nucleus, most delay-period activity encoded the direction of the saccade response [130]. This evidence supports the notion that delay-period activity is important for understanding working memory processes not only in the prefrontal cortex but also in other cortical and subcortical areas.

## 5. Exploring Neural Mechanisms of the Central Executive

Baddeley’s model of working memory is used to explain the results obtained from human psychological and neuropsychological studies and helps us to understand the mechanisms of various cognitive functions including language comprehension, thinking, reasoning, and decision-making. However, since his model is an abstract model, it is difficult to assign each of its components to a particular neural structure or brain area. However, the central executive is a neural component to coordinate the operations of multiple cognitive systems by integrating top-down and bottom-up signals to accomplish a specific goal. These functions are similar to those that have been proposed for the prefrontal cortex. Therefore, the central executive of Baddeley’s model is thought to reflect functions of the prefrontal cortex. In fact, the term “executive function” is often used to describe prefrontal functions. The prefrontal cortex is known to receive a variety of information by bottom-up signaling through cortico-cortical and cortico-subcortical connections and to control the operations of other cortical and subcortical areas by top-down signaling [148,149,150,151,152]. Therefore, it is possible to understand the functions of the central executive by examining the neural mechanisms of prefrontal functions neurophysiologically using primate models.

### 5.1. Neural Mechanisms for Memory Control in the Prefrontal Cortex

As discussed above, Baddeley (2012) [33] proposed a model of working memory, which is composed of one master component (the central executive) and three slave components (the phonological loop, the visuo-spatial sketch pad, and the episodic buffer). The function of the central executive is to coordinate and integrate the operation of the three slave components to achieve a current goal. The dual-task paradigm is often used to examine functions of the central executive [153,154]. The dual-task paradigm is a behavioral procedure in which subjects are required to perform two different tasks simultaneously. When two tasks are performed under the dual-task condition, subjects often exhibit worse performance in one or both of the component tasks compared to when each component task is performed independently. This effect is known as dual-task interference. In human studies, this effect is considered to be caused by a limited capacity of cognitive resource, which corresponds to a task-general information-processing capacity in the brain that is shared by simultaneous cognitive tasks [155,156]. The cognitive resource provides the workspace for information processing in a variety of cognitive functions. However, the capacity of the cognitive resource is limited. Therefore, when the subject needs to perform multiple cognitive tasks simultaneously, some neural system is required to allocate the cognitive resource to each task based on its demand for information processing.

The central executive in Baddeley’s model is thought to be this neural system that allocates a limited capacity of memory to each slave component depending on the demand necessary for its performance. By doing this, the central executive can control the performance of the slave components. Baddeley (1986) [31] did not assign the central executive to any particular brain area. However, shifting of attention or the mental set, updating and monitoring of information, and inhibition of prepotent responses are typical examples of executive functions, and the prefrontal cortex is known to be an essential brain area for such executive functions [157,158,159]. Further, impairment caused by prefrontal damage often produces executive dysfunction [13]. Thus, the functions of the prefrontal cortex are closely linked to the functions of the central executive proposed by Baddeley. Therefore, the prefrontal cortex could act as the central executive and control the operations of other cortical systems. However, it is not yet clear how the prefrontal cortex acts as the central executive and how a prefrontal neural mechanism allocates a limited capacity of memory to each of multiple simultaneous tasks depending on their needs for information processing.

Watanabe and Funahashi (2014, 2015) [160,161] examined this issue by comparing prefrontal neural activities using a dual-task paradigm. They used a spatial working memory task and a spatial attention task. In the spatial working memory task, which is the same as the ODR task, monkeys were required to remember the location of a visual cue that was briefly presented in the peripheral visual field and then perform a memory-guided saccade to that location at the end of the delay period. In the spatial attention task, monkeys were required to attend to a small white circle presented on a monitor and make a quick lever-release response when they detected a change in the color of the circle (from white to red). In the dual-task condition, the spatial working memory task was introduced while monkeys performed the spatial attention task. It is well known that the dorsolateral prefrontal cortex is an essential brain area for both memory and attention tasks [5,79,162] and that many neurons in this area are activated during performance of these tasks [5,57,79,163]. Therefore, simultaneous performance of these two tasks could produce an interference effect, because both tasks require information processing based on a common neural resource. It is expected that, if a dual-task interference effect is observed behaviorally, the delay-period activity observed in the memory task might be affected and there may be a difference in the magnitude of activities between the single-task condition of the memory task and the dual-task condition.

Watanabe and Funahashi (2014, 2015) [160,161] observed that the behavioral performance of the memory task was impaired in the dual-task condition and that greater impairment was observed in the memory task as the difficulty of the attention task increased. Thus, the dual-task interference effect observed in the memory task increased with an increase in the difficulty of the attention task. These results indicate that similar dual-task interference can be observed in both monkeys and human subjects. Next, they analyzed prefrontal single-neuron activities while monkeys performed both the memory task and the attention task under the dual-task condition and while monkeys performed the memory task alone under the single-task condition. They showed that both the memory task and the attention task recruited the activation of largely overlapping prefrontal neural populations. The degree of overlap of the neural populations recruited by these two tasks was correlated with the strength of the dual-task interference effect observed in behavioral studies. A higher degree of overlap in the recruitment of neural populations was observed when a greater interference effect was observed in the behavioral response. In addition, the magnitude of delay-period activity that represented spatial information of the visual cue in the memory task was significantly attenuated in the dual-task condition compared with the single-task condition. Further, a greater attenuation of delay-period activity was observed when the difficulty of the attention task increased in the dual-task condition. These results indicate that the ability of a prefrontal neural population to represent task-relevant information decreases in proportion to the increased demand of the concurrent counterpart task and that the dual-task interference effect originates in the simultaneous and overloaded recruitment of common prefrontal neural populations by the two tasks.

Watanabe and Funahashi (2014) [160] also explored the temporal dynamics of the competitive interaction between the memory task and the attention task in the dual-task condition. When monkeys performed only the attention task, the size of the neural population encoding information regarding the attention cue increased after the attention cue was presented, and its magnitude was maintained until the end of the attention task. Similarly, when monkeys performed only the spatial memory task, the size of the neural population encoding information regarding the memory cue increased after the memory cue was presented, and its magnitude was maintained during the delay period until the end of the memory task. In the dual-task condition, the size of the neural population encoding the attention cue showed similar temporal patterns as when only the attention task was introduced. On the other hand, although the size of the neural population encoding the memory cue transiently increased when the memory cue was presented, it was significantly attenuated during the delay period compared with the single-task condition in the memory task. However, the size of this neural population significantly increased (“reawakening”) at the end of the attention task, and this increase was maintained until the end of the memory task.

These results show that the locus of the dual-task interference effect is the competitive and overloaded recruitment of overlapping neural populations in the prefrontal cortex by the two concurrent tasks. These results also suggest that the capacity of information processing by a single neuron is limited to a fixed level, can be flexibly allocated or reallocated between two concurrent tasks based on their needs, and enhances behavioral performance when allocated to a task. The concurrent elevation, attenuation, and reawakening of the size of neural populations observed in the dual-task condition suggest that the cognitive resource for information processing in the prefrontal cortex is divided and allocated to the two tasks based on their needs.

This view of neural processing has been corroborated by the results of recent single-neuron studies that investigated the neural basis of visual short-term memory for multiple locations [164] and multiple objects [165]. These studies showed that, when two locations or two objects were presented as memoranda within the same visual hemifield, the stimulus selectivity of prefrontal neurons was significantly attenuated relative to when the two stimuli were presented separately in different hemifields. These results suggest that the limited capacity of cognitive resources is the result of the limited computational capacity of single neurons. Dual-task interference is likely to originate in the competitive and overloaded recruitment of common neural populations by two concurrent tasks. Although the neural mechanisms for dividing and allocating a common and limited-capacity cognitive resource in the prefrontal cortex are not known, it is very important to identify these mechanisms when we try to understand the neural mechanisms of executive control by the prefrontal cortex and the neural system that is responsible for the central executive in the working memory model.

### 5.2. Neural Mechanisms for Monitoring Information Contents in the Prefrontal Cortex

In Baddeley’s model of working memory, the central executive plays the most important function among the different components. Miyake et al. (2000) [157] listed three separable executive functions: shifting of attention and the mental set, updating and monitoring of information, and inhibition of prepotent responses. When the prefrontal cortex functions as the central executive by controlling the operations of other cortical and subcortical neural systems using top-down signaling, it must monitor concurrent information processes within itself and other brain areas.

Humans can distinguish between what they remember and do not remember. This ability to monitor one’s own memory state is an important feature of human cognition and is referred to as metamemory [166]. Human neuropsychological studies [167,168] and neuroimaging studies [169,170,171] have revealed that the prefrontal cortex participates in metamemory processes. The neural mechanism of metamemory function is not yet clear, partly because it is difficult to conduct neurobiological studies on metamemory using animals. However, increasing attention has been paid to exploring the neural substrates of metamemory [172,173,174,175]. Neurophysiologists have recently entered this field in the search for the neuronal correlates of uncertainty associated with decision-making [176,177,178,179] and for the neural mechanisms of metamemory itself [180,181,182]. Studies of the neural mechanisms of metamemory functions in the prefrontal cortex could lead to important clues for understanding the neural mechanisms by which the central executive monitors information processes in slave components.

It has been believed that metacognition, which is the ability to monitor and access one’s own memory processes, can only be found in humans, since only humans can use language and express their own introspective experience using language. However, recent behavioral studies have provided evidence that rhesus monkeys can monitor whether or not they remember target information that is necessary to correctly perform subsequent tests [183,184,185]. This ability is thought to be functionally analogous to human metamemory [186]. The metamnemonic ability of monkeys has been examined under an experimental paradigm with two important features. First, difficult memory test conditions are included to ensure that the monkey often experiences uncertainty, such as uncertainty regarding whether or not information that is necessary to make a correct response is remembered. Second, the monkey is given a chance to cope with uncertain trials by selecting an escape option that can be introduced immediately before [184], simultaneously with [183], or immediately after [185] a memory test. This escape option leads to a little more favorable result (e.g., getting a small amount of reward) than failure in the test (e.g., getting no reward), while correct performance on the test yields the most favorable result (e.g., getting a large amount of reward). The basic idea underlying these procedures is that, if the monkey can monitor its own memory state, the monkey can be predicted to select the escape option in trials in which a correct answer is uncertain. Consistent with this prediction, when monkeys perform a memory task that consists of a mixture of trials with various degrees of difficulty, they tend to select the escape option more frequently in more difficult trials, in which they are considered to experience mnemonic uncertainty (lower degree of confidence) more often than in easier trials [183,184,185]. Moreover, by selectively taking the escape option in uncertain trials, monkeys can increase the reward acquisition ratio, compared to when they are required to perform the same task without an escape option (forced choice conditions) [184].

Human studies have indicated that the prefrontal cortex plays an important role in metamemory. The metamnemonic capacity of monkeys can be examined with the use of an appropriate behavior. Neurophysiological studies have shown that prefrontal neurons exhibit directionally-selective delay-period activity, which has been considered to be a neural correlate of a mechanism for the short-term maintenance of spatial information in working memory [1,4,5]. Based on these findings, Tanaka and Funahashi (2012, 2016) [182,187] examined whether and how prefrontal delay-period activity is related to subjective confidence in working memory. Monkeys were trained to perform a modified oculomotor delayed-response task, in which the monkey was required to make a saccade to a memorized location (memory test condition). The difficulty of the memory test was controlled by varying the number of distractors that were presented during the delay period. A feature of this task was the introduction of a choice period between the end of the delay period and the start of the response period. In the choice period, the subject was sometimes allowed to choose to either accept the memory test or decline the test and select the escape option, and sometimes forced to take the memory test. When the subject chose to take the memory test, it was required to make a memory-guided saccade to the location where the visual cue had been presented during the cue period. A correct response was rewarded with a drop of juice, while no reward was given for an incorrect response. When the subject declined the memory test and selected the escape option, it performed a visually-guided saccade to the visual target presented on the monitor, and only 30% to 50% of correct saccades were rewarded. Under these conditions, the monkeys' performance in the memory test condition was significantly better when they chose to take the memory test by themselves than when they were forced to do so [182]. At the same time, the monkeys tended to decline the memory test and chose the escape option more often when the difficulty of the memory test increased [182]. These results agree with the prediction that the monkey can be predicted to select the escape option in trials in which a correct answer is uncertain, and indicate that the monkey can monitor its own working memory state. Middlebrooks and Sommer (2011) [180] also confirmed that monkeys have metamnemonic capacity in working memory.

Tanaka and Funahashi (2016) [187] examined single-neuron activities of prefrontal neurons while monkeys performed this metamemory task. Prefrontal neurons exhibited spatially selective delay-period activity regardless of whether the monkeys chose to take the memory test or to decline the test and take the escape option. However, an important observation was that the spatial selectivity of delay-period activity was significantly weaker when the monkeys chose to decline the memory test than when they chose to take the test. The reduction of spatial selectivity was not due to a decrease in the response to the preferred direction, but rather to an increase in the response to non-preferred directions. Since maintaining spatial information of the visual cue is important for performing this task and since delay-period activity has been considered to be a neural correlate of spatial working memory processes, the directional selectivity of delay-period activity and the maintenance of its strength are essential factors for monkeys to obtain the reward. A decrease in the spatial tuning strength of directional delay-period activity caused a reduction in the accuracy of spatial information regarding the visual cue. Thus, the quality of the spatial information represented by prefrontal delay-period activity is an important factor in the subject's choice regarding whether it chooses to take the memory test or the escape option. Therefore, spatially selective delay-period activity can serve as a source to estimate the subject’s own confidence in their working memory.

On the other hand, Middlebrooks and Sommer (2012) [181] recorded single-neuron activity from the prefrontal cortex, the frontal eye field, and the supplementary eye field while monkeys performed a visual metamemory task using oculomotor responses. Monkeys were required to choose a visual target, which was presented at the same position as where the visual cue had been presented, by a saccade. In this task, monkeys first made a decision and reported it by a saccade. Next, they had to bet whether their decision was correct or not, by selecting one of two visual targets (high-bet target and low-bet target) using a second saccade. If the monkey made a correct decision and took a high-bet target, it obtained the maximum amount of reward. If the monkey made an incorrect decision and took a high-bet target, it received no reward during a 5-s timeout. If the monkey took a low-bet target, it received a small amount of reward regardless of whether it made a correct decision or not. They found that, although neural activity related to the decision and a bet was found in all three brain areas, the activity linking the decision to an appropriate bet was found exclusively in the supplementary eye field. Therefore, they concluded that the supplementary eye field is an important brain area for metacognitive processes and that the putative metacognitive activity began swiftly in the supplementary eye field during the decision stage and continued to the bet stage. Middlebrooks and Sommer (2011, 2012) [180,181] used a task in which the monkeys needed to bet to reflect their confidence in their choice after they made a choice based on their memory. In contrast, Tanaka and Funahashi (2012, 2016) [182,187] used a task in which the monkeys needed to choose a response after they estimated the confidence of their memory. Therefore, the difference between the tasks used may be responsible for the different activities in each brain area and the different contributions of different brain areas. Although these studies found some neural sources of metamemory processes, the neural mechanisms of metamemory processes and monitoring are not yet clear. Further studies are needed to identify which brain areas participate in metamemory processes, how the prefrontal cortex controls metamemory processes and how the prefrontal cortex monitors processes within itself and other areas.

## 6. Conclusions

Delay-period activity is usually defined as tonic sustained activation observed during the delay period. This definition is based on the temporal profile of the neuron’s discharge rate during the delay period. If we use this definition, delay-period activity can be observed not only in the prefrontal cortex but also in any brain area when the subject performs any task with a delay interval. However, since each brain area performs unique information processes using unique information, the information represented by delay-period activity may be different in different brain areas, even if these brain areas show the same temporal profile of delay-period activity. Therefore, a detailed analysis of the features of delay-period activity (e.g., information represented by this activity, selectivity, ratio of neurons having this activity) is critically important for understanding the mnemonic functions and the neural mechanisms of working memory in this brain area.

In the prefrontal cortex, delay-period activity is a neural correlate of the mechanism for temporarily maintaining information and a majority of this activity exhibits stimulus selectivity and encodes retrospective sensory (visual) information. Working memory consists of neural processes not only for the short-term active maintenance of information but also for manipulation of information. When executive functions operated by the prefrontal cortex are considered, neural processes for manipulating information would be more important than for maintaining information. Examining neural activities using a dual-task paradigm or a metamemory task, some important mechanisms related to the central executive have been identified. Although further studies are needed to elucidate how the prefrontal cortex controls memory resource and supervises other systems, detailed analysis of delay-period activity could provide important clue for these mechanisms.

## References

[1] Goldman-Rakic P.S., Plum F. (1987). Circuitry of primate prefrontal cortex and regulation of behavior by representational memory. Higher Functions of the Brain: The Nervous System; Handbook of Physiology.

[2] Miller E.K. (2000). The prefrontal cortex and cognitive control. Nat. Rev. Neurosci..

[3] Miller E.K., Cohen J.D. (2001). An integrative theory of prefrontal cortex function. Annu. Rev. Neurosci..

[4] Fuster J.M. (2008). The Prefrontal Cortex.

[5] Funahashi S. (2015). Functions of delay-period activity in the prefrontal cortex and mnemonic scotomas revisited. Front. Syst. Neurosci..

[6] Jacobson S., Trojanowski J.Q. (1977). Prefrontal granular cortex of the rhesus monkey. I. Intrahemispheric cortical afferents. Brain Res..

[7] Petrides M., Pandya D.N. (1984). Projections to the frontal cortex from the posterior parietal region in the rhesus monkey. J. Comp. Neurol..

[8] Petrides M., Pandya D.N., Boller F., Spinnler H., Hendler J.A. (1994). Comparative architectonic analysis of the human and the macaque frontal cortex. Handbook of Neuropsychology.

[9] Selemon L.D., Goldman-Rakic P.S. (1988). Common cortical and subcortical targets of the dorsolateral prefrontal and posterior parietal cortices in the rhesus monkey: Evidence for a distributed neural network subserving spatially guided behavior. J. Neurosci..

[10] Cavada C., Goldman-Rakic P.S. (1989). Posterior parietal cortex in rhesus monkey: II. Evidence for segregated corticocortical networks linking sensory and limbic areas with the frontal lobe. J. Comp. Neurol..

[11] Goldman-Rakic P.S., Bates J.F., Chafee M.V. (1992). The prefrontal cortex and internally generated motor acts. Curr. Opin. Neurobiol..

[12] Bates J.F., Goldman-Rakic P.S. (1993). Prefrontal connections of medial motor areas in the rhesus monkey. J. Comp. Neurol..

[13] Stuss D.T., Benson D.F. (1986). The Frontal Lobes.

[14] Jacobsen C.F. (1936). Studies of cerebral function in primate. I. The functions of the frontal association areas in monkeys. Comp. Psychol. Monogr..

[15] Rosenkilde C.E. (1979). Functional heterogeneity of the prefrontal cortex in the monkey: A review. Behav. Neural Biol..

[16] Curtis C.E., D’Esposito M. (2004). The effects of prefrontal lesions on working memory performance and theory. Cogn. Affect. Behav. Neurosci..

[17] Goldman-Rakic P.S. (1996). Regional and cellular fractionation of working memory. Proc. Natl. Acad. Sci. USA.

[18] Goldman-Rakic P.S. (1995). Cellular basis of working memory. Neuron.

[19] Baddeley A.D., Hitch G.J., Bower G.S. (1974). Working memory. The Psychology of Learning and Motivation: Advances in Research and Theory.

[20] Mahut H. (1971). Spatial and object reversal learning in monkeys with partial temporal lobe ablations. Neuropsychologia.

[21] O’Keefe J., Nadel L. (1978). The Hippocampus as a Cognitive Map.

[22] Olton D.S., Becker J.T., Handelmann G.E. (1979). Hippocampus, space, and memory. Behav. Brain Sci..

[23] Olton D.S., Papas B. (1979). Spatial memory and hippocampal function. Neuropsychologia.

[24] Nadel L., MacDonald L. (1980). Hippocampus: Cognitive map or working memory?. Behav. Neural Biol..

[25] Aggleton J.P., Hunt P.R., Rawlins J.N.P. (1986). The effects of hippocampal lesions upon spatial and non-spatial tests of working memory. Behav. Brain Res..

[26] Jarrard L.E. (1993). On the role of the hippocampus in learning and memory in the rat. Behav. Neural Biol..

[27] Atkinson R.C., Shiffrin R.M., Spence K.W., Spence J.T. (1968). Human memory: A proposed system and its control processes. The Psychology of Learning and Motivation: Advances in Research and Theory.

[28] Baddeley A.D., Logie R.H., Miyake A., Shah P. (1999). Working memory: The multiple component model. Models of Working Memeory: Mechanisms of Active Maintenance and Executive Control.

[29] Kieras D.E., Meyer D.E., Mueller S., Seymour T., Miyake A., Shah P. (1999). Insights into working memory from the perspective of the EPIC architecture for modeling skilled perceptual-motor and cognitive human performance. Models of Working Memory: Mechanisms of Active Maintenance and Executive Control.

[30] Miyake A., Shah P. (1999). Models of Working Memory: Mechanisms of Active Maintenance and Executive Control.

[31] Baddeley A.D. (1986). Working Memory.

[32] Baddeley A.D. (2000). The episodic buffer: A new component of working memory?. Trends Cogn. Sci..

[33] Baddeley A. (2012). Working memory: Theories, models, and controversies. Annu. Rev. Psychol..

[34] Knowlton B.J., Shapiro M.L., Olton D.S. (1989). Hippocampal seizures disrupt working memory performance but not reference memory acquisition. Behav. Neurosci..

[35] Stokes K.A., Best P.J. (1990). Mediodorsal thalamic lesions impair “reference” and “working” memory in rats. Physiol. Behav..

[36] Ando S., Ohashi Y. (1991). Longitudinal study on age-related changes of working and reference memory in the rat. Neurosci. Lett..

[37] Sakurai Y. (1992). Auditory working and reference memory can be tested in a single situation of stimuli for the rat. Behav. Brain Res..

[38] Bushnell P.J., Levin E.D. (1993). Effects of dopaminergic drugs on working and reference memory in rats. Pharmacol. Biochem. Behav..

[39] Prior H., Schwegler H., Ducker G. (1997). Dissociation of spatial reference memory, spatial working memory, and hippocampal mossy fiber distribution in two rat strains differing in emotionality. Behav. Brain Res..

[40] Gresack J.E., Frick K.M. (2003). Male mice exhibit better spatial working and reference memory than females in a water-escape radial arm maze task. Brain Res..

[41] Honig W.K., Hulse S.H., Fowler H., Honig W.K. (1978). Studies of working memory in the pigeon. Cognitive Processes in Animal Behavior.

[42] Funahashi S. (2013). Space representation in the prefrontal cortex. Prog. Neurobiol..

[43] Raffaele K.C., Olton D.S. (1988). Hippocampal and Amygdaloid involvement in working memory for nonspatial stimuli. Behav. Neurosci..

[44] Bachevalier J., Mishkin M. (1986). Visual recognition impairment follows ventromedial but not dorsolateral prefrontal lesions in monkeys. Behav. Brain Res..

[45] Miller E.K., Li L., Desimone R. (1991). A neural mechanism for working and recognition memory in inferior temporal cortex. Science.

[46] Overman W., Bachevalier J., Turner M., Peuster A. (1992). Object recognition versus object discrimination: Comparison between human infants and infant monkeys. Behav. Neurosci..

[47] Zola S.M., Squire L.R., Teng E., Stefanacci L., Buffalo E.A., Clark R.E. (2000). Impaired recognition memory in monkeys after damage limited to the hippocampal region. J. Neurosci..

[48] Brown M.W., Aggleton J.P. (2001). Recognition memory: What are the roles of the perirhinal cortex and hippocampus?. Nat. Rev. Neurosci..

[49] Squire L.R., Wixted J.T., Clark R.E. (2007). Recognition memory and the medial temporal lobe: A new perspective. Nat. Rev. Neurosci..

[50] Eichenbaum H., Yonelinas A.P., Ranganath C. (2007). The medial temporal lobe and recognition memory. Annu. Rev. Neurosci..

[51] Butters N., Pandya D. (1969). Retention of delayed alternation: Effect of selective lesions of sulcus principalis. Science.

[52] Malmo R.B. (1942). Interference factors in delayed response in monkeys after removal of frontal lobes. J. Neurophysiol..

[53] Bartus R.T., Levere T.E. (1977). Frontal decortication in rhesus monkeys: A test of the interference hypothesis. Brain Res..

[54] Funahashi S., Bruce C.J., Goldman-Rakic P.S. (1993). Dorsolateral prefrontal lesions and oculomotor delayed response performance: Evidence for mnemonic “scotomas”. J. Neurosci..

[55] Kubota K., Niki H. (1971). Prefrontal cortical unit activity and delayed alternation performance in monkeys. J. Neurophysiol..

[56] Fuster J.M., Alexander G.E. (1971). Neuron activity related to short-term memory. Science.

[57] Fuster J.M. (1973). Unit activity in prefrontal cortex during delayed-response performance: Neuronal correlates of transient memory. J. Neurophysiol..

[58] Niki H. (1974). Differential activity of prefrontal units during right and left delayed response trials. Brain Res..

[59] Niki H., Watanabe M. (1976). Prefrontal unit activity and delayed response: Relation to cue location versus direction of response. Brain Res..

[60] Niki H. (1974). Prefrontal unit activity during delayed alternation in the monkey. I. Relation to direction of response. Brain Res..

[61] Niki H. (1974). Prefrontal unit activity during delayed alternation in the monkey. II. Relation to absolute versus relative direction of response. Brain Res..

[62] Kojima S., Goldman-Rakic P.S. (1982). Delay-related activity of prefrontal neurons in rhesus monkeys performing delayed response. Brain Res..

[63] Kojima S., Goldman-Rakic P.S. (1984). Functional analysis of spatially discriminative neurons in prefrontal cortex of rhesus monkey. Brain Res..

[64] Carlson S., Rama P., Tanila H., Linnankoski I., Mansikka H. (1997). Dissociation of mnemonic coding and other functional neuronal processing in the monkey prefrontal cortex. J. Neurophysiol..

[65] Carlson S., Tanila H., Pertovaara A., Lahteenmaki A. (1990). Vertical and horizontal coding of space in the monkey dorsolateral prefrontal cortex. Brain Res..

[66] Funahashi S., Inoue M., Kubota K. (1997). Delay-period activity in the primate prefrontal cortex encoding multiple spatial positions and their order of presentation. Behav. Brain Res..

[67] Joseph J.P., Barone P. (1987). Prefrontal unit activity during a delayed oculomotor task in the monkey. Exp. Brain Res..

[68] Barone P., Joseph J.P. (1989). Prefrontal cortex and spatial sequencing in macaque monkey. Exp. Brain Res..

[69] Boch R.A., Goldberg M.E. (1989). Participation of prefrontal neurons in the preparation of visually guided eye movements in the rhesus monkey. J. Neurophysiol..

[70] Funahashi S., Bruce C.J., Goldman-Rakic P.S. (1991). Neuronal activity related to saccadic eye movements in the monkey’s dorsolateral prefrontal cortex. J. Neurophysiol..

[71] Funahashi S. (2014). Saccade-related activity in the prefrontal cortex: Its role in eye movement control and cognitive functions. Front. Integr. Neurosci..

[72] Suzuki H., Azuma M. (1977). Prefrontal neuronal activity during gazing at a light spot in the monkey. Brain Res..

[73] Suzuki H., Azuma M., Yumiya H. (1979). Stimulus and behavioral factors contributing to the activation of monkey prefrontal neurons during gazing. Jpn. J. Physiol..

[74] Boussaoud D., Barth T.M., Wise S.P. (1993). Effects of gaze on apparent visual responses of frontal cortex neurons. Exp. Brain Res..

[75] Andersen R.A., Essick G.K., Siegel R.M. (1985). Encoding of spatial location by posterior parietal neurons. Science.

[76] Andersen R.A., Mountcastle V.B. (1983). The influence of the angle of gaze upon the excitability of the light-sensitive neurons of the posterior parietal cortex. J. Neurosci..

[77] Andersen R.A., Snyder L.H., Bradley D.C., Xing J. (1997). Multimodal representation of space in the posterior parietal cortex and its use in planning movements. Annu. Rev. Neurosci..

[78] Squatrito S., Maioli M.G. (1996). Gaze field properties of eye position neurons in area MST and 7a of the macaque monkey. Vis. Neurosci..

[79] Funahashi S., Bruce C.J., Goldman-Rakic P.S. (1989). Mnemonic coding of visual space in the monkey's dorsolateral prefrontal cortex. J. Neurophysiol..

[80] Wilson F.A.W., Scalaidhe S.P., Goldman-Rakic P.S. (1993). Dissociation of object and spatial processing domains in primate prefrontal cortex. Science.

[81] Williams G.V., Goldman-Rakic P.S. (1995). Modulation of memory fields by dopamine D1 receptors in prefrontal cortex. Nature.

[82] Hasegawa R., Sawaguchi T., Kubota K. (1998). Monkey prefrontal neuronal activity coding the forthcoming saccade in an oculomotor delayed matching-to-sample task. J. Neurophysiol..

[83] Chafee M.V., Goldman-Rakic P.S. (1998). Matching patterns of activity in primate prefrontal area 8s and parietal area 7ip neurons during a spatial working memory. J. Neurophysiol..

[84] Chafee M.V., Goldman-Rakic P.S. (2000). Inactivation of parietal and prefrontal cortex reveals interdependence of neural activity during memory-guided saccades. J. Neurophysiol..

[85] Constantinidis C., Franowicz M.N., Goldman-Rakic P.S. (2001). The sensory nature of mnemonic representation in the primate prefrontal cortex. Nat. Neurosci..

[86] Constantinidis C., Franowicz M.N., Goldman-Rakic P.S. (2001). Coding specificity in cortical microcircuits: A multiple-electrode analysis of primate prefrontal cortex. J. Neurosci..

[87] Sawaguchi T., Iba M. (2001). Prefrontal cortical representation of visuospatial working memory in monkeys examined by local inactivation with muscimol. J. Neurophysiol..

[88] Takeda K., Funahashi S. (2002). Prefrontal task-related activity representing visual cue location or saccade direction in spatial working memory tasks. J. Neurophysiol..

[89] Williams G.V., Rao S.G., Goldman-Rakic P.S. (2002). The physiological role of 5HT_2A_ receptors in working memory. J. Neurosci..

[90] Tsujimoto S., Sawaguchi T. (2004). Properties of delay-period neuronal activity in the primate prefrontal cortex during memory- and sensory-guided saccade tasks. Eur. J. Neurosci..

[91] Rainer G., Asaad W.F., Miller E.K. (1998). Memory fields of neurons in the primate prefrontal cortex. Proc. Natl. Acad. Sci. USA.

[92] Funahashi S., Chafee M.V., Goldman-Rakic P.S. (1993). Prefrontal neuronal activity in rhesus monkeys performing a delayed anti-saccade task. Nature.

[93] Sawaguchi T., Yamane I. (1999). Properties of delay-period neuronal activity in the monkey dorsolateral prefrontal cortex during a spatial delayed matching-to-sample task. J. Neurophysiol..

[94] Gnadt J.W., Andersen R.A. (1988). Memory related motor planning activity in posterior parietal cortex of macaque. Exp. Brain Res..

[95] Crammond D.J., Kalaska J.F. (1989). Neuronal activity in primate parietal cortex area 5 varies with intended movement direction during an instructed-delay period. Exp. Brain Res..

[96] Koch K.W., Fuster J.M. (1989). Unit activity in monkey parietal cortex related to haptic perception and temporary memory. Exp. Brain Res..

[97] Constantinidis C., Steinmetz M.A. (1996). Neuronal activity in posterior parietal area 7a during the delay periods of a spatial memory task. J. Neurophysiol..

[98] Snyder L.H., Batista A.P., Andersen R.A. (1997). Coding of intention in the posterior parietal cortex. Nature.

[99] Quintana J., Fuster J.M. (1999). From perception to action: Temporal integrative functions of prefrontal and parietal neurons. Cereb. Cortex.

[100] Calton J.L., Dickinson A.R., Snyder L.H. (2002). Non-spatial, motor-specific activation in posterior parietal cortex. Nat. Neurosci..

[101] Pesaran B., Pezaris J.S., Sahani M., Mitra P.P., Andersen R.A. (2002). Temporal structure in neuronal activity during working memory in macaque parietal cortex. Nat. Neurosci..

[102] Huk A.C., Shadlen M.N. (2005). Neural activity in macaque parietal cortex reflects temporal integration of visual motion signals during perceptual decision making. J. Neurosci..

[103] Nieder A., Diester I., Tudusciuc O. (2006). Temporal and spatial enumeration processes in the primate parietal cortex. Science.

[104] Tudusciuc O., Nieder A. (2007). Neuronal population coding of continuous and discrete quantity in the primate posterior parietal cortex. Proc. Natl. Acad. Sci. USA.

[105] Katsuki F., Constantinidis C. (2012). Unique and shared roles of the posterior parietal and dorsolateral prefrontal cortex in cognitive functions. Front. Integr. Neurosci..

[106] Fuster J.M., Jervey J.P. (1982). Neuronal firing in the inferotemporal cortex of the monkey in a visual memory task. J. Neurosci..

[107] Miyashita Y. (1988). Neuronal correlate of visual associative long-term memory in the primate temporal cortex. Nature.

[108] Miyashita Y., Chang H.S. (1988). Neuronal correlate of pictorial short-term memory in the primate temporal cortex. Nature.

[109] Sakai K., Miyashita Y. (1991). Neural organization for the long-term memory of paired associates. Nature.

[110] Miller E.K., Li L., Desimone R. (1993). Activity of neurons in anterior inferior temporal cortex during a short-term memory task. J. Neurosci..

[111] Chelazzi L., Duncan J., Miller E.K., Desimone R. (1998). Responses of neurons in inferior temporal cortex during memory-guided visual search. J. Neurophysiol..

[112] Yakovlev V., Fisi S., Berman E., Zohary E. (1998). Inter-trial neuronal activity in inferior temporal cortex: A putative vehicle to generate long-term visual associations. Nat. Neurosci..

[113] Zhou Y.-D., Fuster J.M. (1996). Mnemonic neuronal activity in somatosensory cortex. Proc. Natl. Acad. Sci. USA.

[114] Zhou Y.-D., Fuster J.M. (1997). Neuronal activity of somatosensory cortex in a cross-modal (visuo-haptic) memory task. Exp. Brain Res..

[115] Weinrich M., Wise S.P. (1982). The premotor cortex of the monkey. J. Neurosci..

[116] Kurata K., Wise S.P. (1988). Premotor cortex of rhesus monkeys: Set-related activity during two conditional motor tasks. Exp. Brain Res..

[117] Crammond D.J., Kalaska J.F. (2000). Prior information in motor and premotor cortex: Activity during the delay period and effect on pre-movement activity. J. Neurophysiol..

[118] Ohbayashi M., Ohki K., Miyashita Y. (2003). Conversion of working memory to motor sequence in the monkey premotor cortex. Science.

[119] Gibson J.R., Maunsell J.H.R. (1997). Sensory modality specificity of neural activity related to memory in visual cortex. J. Neurophysiol..

[120] Lee H., Simpson G.V., Logothetis N.K., Rainer G. (2006). Phase locking of single neuron activity to theta oscillations during working memory in monkey extrastriate visual cortex. Neuron.

[121] Hikosaka O., Wurtz R.H. (1983). Visual and oculomotor functions of monkey substantia nigra pars reticulate. III. Memory-contingent visual and saccade responses. J. Neurophysiol..

[122] Basso M.A., Wurtz R.H. (1998). Modulation of neuronal activity in superior colliculus by changes in target propability. J. Neurosci..

[123] Niki H., Sakai M., Kubota K. (1972). Delayed alternation performance and unit activity of the caudate head and medial orbitofrontal gyrus in the monkey. Brain Res..

[124] Soltysik S., Hull C.D., Buchwald N.A., Fekete T. (1975). Single unit activity in basal ganglia of monkeys during performance of a delayed response task. Electroencephalogr. Clin. Neurophysiol..

[125] Hikosaka O., Sakamoto M., Usui S. (1989). Functional properties of monkey caudate neurons. III. Activities related to expectation of target and reward. J. Neurophysiol..

[126] Apicella P., Scarnati E., Ljungberg T., Schultz W. (1992). Neuronal activity in monkey striatum related to the expectation of predictable environmental events. J. Neurophysiol..

[127] Watanabe T., Niki H. (1985). Hippocampal unit activity and delayed response in the monkey. Brain Res..

[128] Riches I.P., Wilson F.A.W., Brown M.W. (1991). The effects of visual stimulation and memory on neurons of the hippocampal formation and the neighboring parahippocampal gyrus and inferior temporal cortex of the primate. J. Neurosci..

[129] Watanabe Y., Funahashi S. (2004). Neuronal activity throughout the primate mediodorsal nucleus of the thalamus during oculomotor delayed-response. I. Cue-, delay-, and response-period activity. J. Neurophysiol..

[130] Watanabe Y., Funahashi S. (2004). Neuronal activity throughout the primate mediodorsal nucleus of the thalamus during oculomotor delayed-response. II. Activity encoding visual versus motor signal. J. Neurophysiol..

[131] Prut Y., Fetz E.E. (1999). Primate spinal interneurons show re-movement instructed delay activity. Nature.

[132] Romo R., Brody C.D., Hernandez A., Lemus L. (1999). Neuronal correlates of parametric working memory in the prefrontal cortex. Nature.

[133] Kikuchi-Yorioka Y., Sawaguchi T. (2000). Parallel visuospatial and audiospatial working memory processes in the monkey dorsolateral prefrontal cortex. Nat. Neurosci..

[134] White I.M., Wise S.P. (1999). Rule-dependent neuronal activity in the prefrontal cortex. Exp. Brain Res..

[135] Hoshi E., Shima K., Tanji J. (2000). Neuronal activity in the primate prefrontal cortex in the process of motor selection based on two behavioral rules. J. Neurophysiol..

[136] Wallis J.D., Anderson K.C., Miller E.K. (2001). Single neurons in prefrontal cortex encode abstract rules. Nature.

[137] Amemori K., Sawaguchi T. (2006). Rule-dependent shifting of sensorimotor representation in the primate prefrontal cortex. Eur. J. Neurosci..

[138] Asaad W.F., Rainer G., Miller E.K. (2000). Task-specific neural activity in the primate prefrontal cortex. J. Neurophysiol..

[139] Watanabe M. (1996). Reward expectancy in primate prefrontal neurons. Nature.

[140] Leon M.I., Shadlen M.N. (1999). Effects of expected reward magnitude on the response of neurons in the dorsolateral prefrontal cortex of the macaque. Neuron.

[141] Kobayashi S., Lauwereyns J., Koizumi M., Sakagami M., Hikosaka O. (2002). Influence of reward expectation on visuospatial processing in macaque lateral prefrontal cortex. J. Neurophysiol..

[142] Watanabe M., Hikosaka K., Sakagami M., Shirakawa S. (2002). Coding and monitoring of motivational context in the primate prefrontal cortex. J. Neurosci..

[143] Nieder A., Freedman D.J., Miller E.K. (2002). Representation of the quantity of visual items in the primate prefrontal cortex. Science.

[144] Nieder A., Miller E.K. (2003). Coding of cognitive magnitude: Compressed scaling of numerical information in the primate prefrontal cortex. Neuron.

[145] Genovesio A., Tsujimoto S., Wise S.P. (2011). Prefrontal cortex activity during the discrimination of relative distance. J. Neurosci..

[146] Genovesio A., Tsujimoto S., Wise S.P. (2009). Feature- and order-based timing representations in the frontal cortex. Neuron.

[147] Gottlieb J.P., Kusunoki M., Goldberg M.E. (1998). The representation of visual salience in monkey parietal cortex. Nature.

[148] Tomita H., Ohbayashi M., Nakahara K., Hasegawa I., Miyashita Y. (1999). Top-down signal from prefrontal cortex in executive control of memory retrieval. Nature.

[149] Johnstone T., van Reekum C.M., Urry H.L., Kalin N.H., Davidson R.J. (2007). Failure to regulate: Counterproductive recruitment of top-down prefrontal-subcortical circuitry in major depression. J. Neurosci..

[150] Hwang K., Velanova K., Luna B. (2010). Strengthening of top-down frontal cognitive control networks underlying the development of inhibitory control: A functional magnetic resonance imaging effective connectivity study. J. Neurosci..

[151] Lee T.G., D’Esposito M. (2012). The dynamic nature of top-down signals originating from prefrontal cortex: A combined fMRI-TMS study. J. Neurosci..

[152] Funahashi S., Andreau J.M. (2013). Prefrontal cortex and neural mechanisms of executive function. J. Physiol. Paris.

[153] Just M.A., Carpenter P.A. (1992). A capacity theory of comprehension: Individual differences in working memory. Psychol. Rev..

[154] Baddeley A.D. (1996). Exploring the central executive. Q. J. Exp. Psychol..

[155] Moray N. (1967). Where is capacity limited? A survey and a model. Acta Psychol..

[156] Wickens C.D., Nickerson R.S. (1980). The structure of attentional resources. Attention and Performance VIII.

[157] Miyake A., Friedman N.P., Emerson M.J., Witzki A.H., Howerter A. (2000). The unity and diversity of executive functions and their contributions to complex “frontal lobe” tasks: A latent variable analysis. Cogn. Psychol..

[158] Stuss D.T., Alexander M.P. (2000). Executive functions and the frontal lobes: A conceptual view. Psychol. Res..

[159] Alvarez J.A., Emory E. (2006). Executive function and the frontal lobes: A meta-analytic review. Neuropsychol. Rev..

[160] Watanabe K., Funahashi S. (2014). Neural mechanisms of dual-task interference and cognitive capacity limitation in the prefrontal cortex. Nat. Neurosci..

[161] Watanabe K., Funahashi S. (2015). A dual-task paradigm for behavioral and neurobiological studies in nonhuman primates. J. Neurosci. Methods.

[162] Rossi A.F., Bichot N.P., Desimone R., Ungerleider L.G. (2007). Top-down attentional deficits in macaques with lesions of lateral prefrontal cortex. J. Neurosci..

[163] Kadohisa M., Petrov P., Stokes M., Sigala N., Buckley M., Gaffan D., Kusunoki M., Duncan J. (2013). Dynamic construction of a coherent attentional state in a prefrontal cell population. Neuron.

[164] Matsushima A., Tanaka M. (2014). Different neuronal computations of spatial working memory for multiple locations within versus across visual hemifields. J. Neurosci..

[165] Buschman T.J., Siegel M., Roy J.E., Miller E.K. (2011). Neural substrates of cognitive capacity limitations. Proc. Natl. Acad. Sci. USA.

[166] Nelson T.O., Narens L., Bower G. (1990). Metamemory: A theoretical framework and new findings. The Psychology of Learning and Motivation: Advances in Research and Theory.

[167] Janowsky J.S., Shimamura A.P., Squire L.R. (1989). Memory and metamemory: Comparisons between patients with frontal lobe lesions and amnesiac patients. Psychobiology.

[168] Schnyer D., Verfaellie M., Alexander M., LaFleche G., Nicholls L., Kaszniak A.W. (2004). A role for right medial prefrontal cortex in accurate feeling-of-knowing judgments: Evidence from patients with lesions to frontal cortex. Neuropsychologia.

[169] Kikyo H., Ohki K., Miyashita Y. (2002). Neural correlates for feeling-of-knowing: An fMRI parametric analysis. Neuron.

[170] Maril A., Simons J.S., Mitchell J.P., Schwartz B.L., Schacter D.L. (2003). Feeling-of-knowing in episodic memory: An event-related fMRI study. Neuroimage.

[171] Schnyer D.M., Nicholls L., Verfaellie M. (2005). The role of VMPC in metamemorial judgments of content retrievability. J. Cogn. Neurosci..

[172] Pannu J.K., Kaszniak A.W. (2005). Metamemory experiments in neurological populations: A review. Neuropsychol. Rev..

[173] Schwartz B.L., Bacon E., Dunlosky J., Bjork R.A. (2008). Metacognitive neuroscience. Handbook of Metamemory and Memory.

[174] Shimamura A.P., Dunlosky J., Bjork R.A. (2008). A neurocognitive approach to metacognitive monitoring and control. Handbook of Metamemory and Memory.

[175] Fleming S.M., Lau H.C. (2014). How to measure metacognition. Front. Hum. Neurosci..

[176] Kepecs A., Uchida N., Zariwala H., Mainen Z.F. (2008). Neural correlates, computation and behavioural impact of decision confidence. Nature.

[177] Kiani R., Shadlen M.N. (2009). Representation of confidence associated with a decision by neurons in the parietal cortex. Science.

[178] Fleming S.M., Huijgen J., Dolan R.J. (2012). Prefrontal contribution to metacognition in perceptual decision making. J. Neurosci..

[179] Komura Y., Nikkuni A., Hirashima N., Uetake T., Miyamoto A. (2013). Responses of pulvinar neurons reflect a subject’s confidence in visual categorization. Nat. Neurosci..

[180] Middlebrooks P.G., Sommer M.A. (2011). Metacognition in monkeys during an oculomotor task. J. Exp. Psychol. Learn. Mem. Cogn..

[181] Middlebrooks P.G., Sommer M.A. (2012). Neuronal correlates of metacognition in primate frontal cortex. Neuron.

[182] Tanaka A., Funahashi S. (2012). Macaque monkeys exhibit behavioral signs of metamemory in an oculomotor working memory task. Behav. Brain Res..

[183] Smith J.D., Shields W.E., Allendoerfer K.R., Washburn D.A. (1998). Memory monitoring by animals and humans. J. Exp. Psychol. Gen..

[184] Hampton R.R. (2001). Rhesus monkeys know when they remember. Proc. Natl. Acad. Sci. USA.

[185] Kornell N., Son L.K., Terrace H.S. (2007). Transfer of metacognitive skills and hint seeking in monkeys. Psychol. Sci..

[186] Smith J.D., Shields W.E., Washburn D.A. (2003). The comparative psychology of uncertainty monitoring and metacognition. Behav. Brain Sci..

[187] Tanaka A., Funahashi S. (2016). Persistent activity of prefrontal neurons as a source of confidence in working memory. 2016 Neuroscience Meeting Planner, Program No. 550.01, Online.

